# Evaluation of Contralateral Limb Cross Education and High-Frequency Repetitive Transcranial Magnetic Stimulation on Functional Indices of the Affected Upper Limb in Subacute Phase of Stroke

**DOI:** 10.1155/2023/4387667

**Published:** 2023-12-19

**Authors:** Katayoon Rezaei, Amin Kordi Yoosefinejad, Farzaneh Moslemi Haghighi, Mohsen Razeghi

**Affiliations:** ^1^Department of Physical Therapy, School of Rehabilitation Sciences, Shiraz University of Medical Sciences, Shiraz, Iran; ^2^Rehabilitation Sciences Research Center, Shiraz University of Medical Sciences, Shiraz, Iran

## Abstract

**Background:**

Stroke is one of the causes of long-term morbidity. Despite rehabilitation strategies, most survivors live with motor deficits in the upper limbs.

**Objectives:**

The aim of the study was to compare the effect of contralateral cross education (CE) and high-frequency repetitive magnetic stimulation (HF-rTMS) on the function of upper extremity in subacute phase of stroke.

**Methods:**

Forty patients were randomly assigned into 4 groups. Group “A” received physical therapy (PT) for 10 sessions, 3 times per week. Group “B” received PT and HF-rTMS as follows: stimulation of 20 Hz for 5 s, intertrain interval for 50 s, 20 trains, 2000 pulses at 90% resting motor threshold, and conventional PT. Group “C” was treated with CE and PT. In group “D,” HF-rTMS, CE, and PT were administered.

**Results:**

Significant differences were found in the Fugl-Meyer scale between “A” and “C” (*P* = 0.01), “A” and “D” (*P* = 0.02), and “B” and “C” groups (*P* = 0.01). In the box-block test, there were significant differences between “A” and “B” (*P* = 0.01), “A” and “C” (*P* < 0.001), “B” and “D” (*P* = 0.001), and “B” and “C” groups (*P* = 0.01). Statistical differences were observed in grip strength between “A” and “B” (*P* = 0.01) and “A” and “C” groups (*P* = 0.02).

**Conclusions:**

It is suggested that clinicians select the therapeutic methods in line with their expected goal. When the goal is to improve upper extremity function, CE+PT could be more effective than HF-rTMS+PT. Also, CE+PT and HF-rTMS+PT were more effective than CE+HF-rTMS+PT at improving grip strength. Therefore, combining several methods would not always lead to better results.

## 1. Introduction

Stroke is considered as the major cause of long-term motor disability in adults [1, 2]. Despite using intensive rehabilitation strategies, most survivors suffer from motor deficits especially in the upper limbs [3, 4]. Previous studies have reported that only 60% of patients with hemiparesis would experience functional independence in their routine daily activities [3, 5].

The neural mechanisms underlying poststroke functional recovery are yet to be known. Several neuroimaging studies have shown that motor recovery following stroke could be attributed to neuroplasticity and cortical reorganization [6]. Recently, noninvasive brain stimulation methods, such as repetitive transcranial magnetic stimulation (rTMS), have been used to modulate cortical excitability to induce neuroplasticity. The beneficial effects of rTMS on poststroke motor deficits are based upon interhemispheric competition model [7]. In healthy individuals, neural activity of motor areas of each hemisphere is functionally coupled with the other hemisphere and is equally balanced [8, 9]. Following a stroke, the interhemispheric symmetry of brain and the cortical excitability have altered [10]. Sharma et al. showed that applying low-frequency rTMS on the contralesional premotor cortex along with conventional physical therapy significantly improved modified Barthel index in patients with subacute ischemic stroke [11].

In accordance with the interhemispheric competition model, the “overactive” motor areas of the contralesioned hemisphere impose excessive inhibition on the lesioned hemisphere, which may decrease excitability and cortical drive to the paretic extremity [12, 13]. Brain-imaging studies have confirmed that excitatory (i.e., high frequency) rTMS on the lesioned hemisphere and inhibitory (i.e., low frequency) rTMS on the unlesioned hemisphere can improve equilibrium of cortical excitability between the two hemispheres [14]. A recent meta-analysis provided robust evidence for the efficacy of rTMS at improving upper extremity function during various phases of stroke [15].

Adding rTMS to conventional therapy had beneficial effects for patients with stroke. Luk et al. showed that using low-frequency rTMS prior to motor task practice had reduced interhemispheric asymmetry of cortical excitabilities and promoted upper limb function recovery in patients with subacute stroke [16].

The other method to improve motor recovery, based on interhemispheric interaction, was suggested by basic evidence in healthy individuals and clinical studies. The method is known as cross education (CE), defined as the improved performance of untrained limb following unilateral training of the opposite homologous limb. The underlying mechanisms for interlimb neural circuit are not clearly understood. Studies have shown that CE induces some form of neural adaptive plasticity to both trained and untrained limbs [17]. CE as an adjunctive treatment with transcutaneous electrical nerve stimulation had improved motor function of the untrained upper extremity in patients with stroke [18]. In a recent review incorporating 226 patients with stroke, the beneficial effects of CE on the strength and motor function of more affected upper limb was confirmed [19]. It has been reported that the primary motor cortex (M1) of the two hemispheres interacts during unilateral motor training through corpus callosum pathways [20].

It has been reported that both of these methods are based on interhemispheric interaction and transcallosal pathways [21, 22]. Previous studies suggested that these methods improve functional ability of the hemiparetic patients; however, more studies are warranted to clarify the involved mechanisms. Basic studies have suggested that CE can be used in patients with unilateral impairment. However, most of these studies are performed to assess the effect of CE on motor function in healthy individuals, and there is a dearth of evidence on patients with unilateral deficit. Despite previous suggestions that CE is efficacious in patients with stroke, this method has not been investigated in a clinical setting. Moreover, previous studies showed that the benefits of rTMS in patients with stroke were modest and inconclusive [23, 24]. The combination of rTMS with specific neurorehabilitation methods and occupational therapy was reported to improve motor function [25, 26].

Due to the worldwide increasing number of strokes predicted for 2030 [27], there is an urgent need to develop new strategies aimed to increase the efficacy of treatment, improve the quality of rehabilitation care, and mitigate patients' disability. The objective of this study was to investigate the efficacy of treatment protocols to improve motor function in stroke patients and to compare the potential benefits of designed protocols following unilateral motor impairment in patients at the subacute phase of stroke. Our hypothesis was that rTMS and CE could be incorporated into contemporary conventional rehabilitation programs to accelerate recovery.

## 2. Patients and Methods

It was a parallel-designed RCT study conducted between August 2016 and April 2017 in Rehabilitation Research center, Shiraz University of Medical Sciences, Shiraz, Iran (IRCT number: IRCT2016011726056N1). To determine the sample size, we used the data of a previous study [28] investigating high-frequency (HF) rTMS for the treatment of motor deficit in subacute stroke patients. Considering a power of 80% and a critical alpha = 0.05%, a minimum of twenty-four patients (six in each group) were recruited to be assigned into four groups. To account for possible dropouts (attrition rate of 40%), 40 patients were entered into the study.

**Figure 1:**
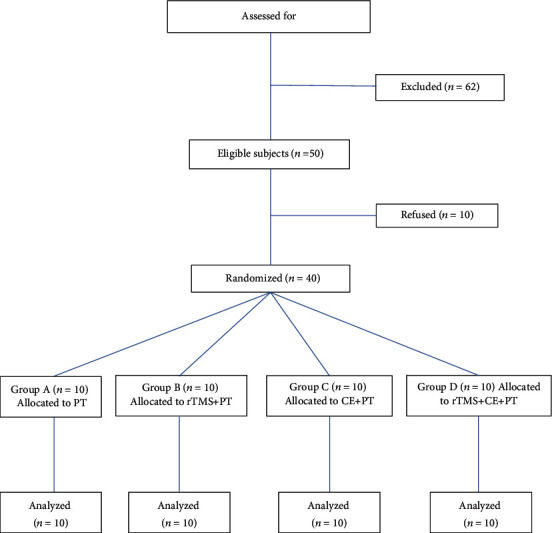
CONSORT flowchart of stroke patients recruited in this study. Abbreviations: rTMS: repetitive transcranial magnetic stimulation; PT: physiotherapy; CE: cross education.

One hundred and twelve patients in the subacute phase of stroke were evaluated consecutively for participating in the study. All patients were admitted to the rehabilitation centers affiliated with Shiraz University of Medical Sciences. The diagnosis was in accordance with the diagnostic criteria established by the Global Academic Conference on Cerebrovascular Disease. The inclusion criteria were as follows: (1) first unilateral stroke within 1 to 6 months from onset, (2) aged 30-65 years, (3) score of 22-44 on the Fugl-Meyer assessment for upper extremity (FMA-UE), and (4) ability to complete the study protocol with both lesioned and unlesioned upper limbs. The exclusion criteria were as follows: (1) other neurological diseases; (2) history of seizure; (3) uncorrected visual field defects; (4) aphasia, ipsilateral neglect, hemianopia, or affective disorders that could affect patient's ability to comply with study procedure; (5) cognitive impairments based on the Mini-Mental State Examination (MMSE ≤ 20/30); (6) recent fracture, dislocation, or subluxation in the upper limbs; (7) rheumatic disorders involving the upper limbs; and (8) absolute and relative risk factors for rTMS [29, 30]. The flowchart of the recruitment process is illustrated in Figure 1. All the enrolled patients provided written consent before the study began. The study was approved by local ethics committee of Vice chancellor for Research, Shiraz University of Medical Sciences (ethics number: 92-7605) in accordance with the standards of Helsinki Declaration.

### 2.1. Study Design

Permuted block randomization (size of block: 4, number of blocks: 10) was used to assign the participants into four groups: conventional physiotherapy (PT) group (group A), rTMS+PT group (group B), CE+PT group (group C), and rTMS+CE+PT group (group D).

Each patient received 10 treatment sessions, three times per week. All the participants were treated by a well-trained and qualified physiotherapist (FMH) with more than 18 years of experience in neurological rehabilitation. The evaluation process was carried out by an experienced physical therapist (K.R) who was blind to group assignment during two separate sessions: one day prior to interventions and one day after the completion of interventions.

### 2.2. Assessments

The outcome measures were FMA-UE, box and block test (BBT), grip strength, and pinch strength. FMA-UE assess the motor function of the affected upper limb. The scale comprises 33 quantitative tasks, and each task is scored from zero (no performance) to two (complete performance) [31]. The maximum motor performance score (66 points) represents normal motor function of the upper extremity. The reliability and validity of this test have been previously confirmed [32].

The BBT was chosen to evaluate gross manual dexterity of the involved upper limb. This test consists of a partitioned box and 150 blocks. The patient must transfer as many blocks as possible from one side to the other side of the box. The number of blocks transferred within 60 seconds is scored. Reliability and validity of this instrument has been established previously [33].

Maximal isometric grip strength was measured in both upper limbs with a handheld grip dynamometer (SEHAN, Masan, Korea). Hydraulic pinch gauge (Model SH5005, SEHAN, Masan, Korea) was used to measure the pinch strength. The mean value of three trials was recorded and measured in kilograms.

### 2.3. Therapeutic Interventions

Group A received conventional PT treatment for 10 sessions, 3 times a week. Depending on the individual's level of ability, based on FMA-UE scale, PT program included joints' range of motion exercises, gentle stretching of hypertonic (spastic) muscles, strengthening of antagonist muscle pattern, activities of daily living training, and functional electrical stimulation (frequency of 25 Hz 250 *μ*s pulse width to achieve controlled muscle contraction, duration of 20 min) [34].

Group B received HF-rTMS and conventional PT. A 70 mm figure-of-eight coil and a Magstim Rapid stimulation (SM9000, Neurosoft, Russia) were used for HF-rTMS. The coil was placed on the affected motor cortex. Prior to rTMS stimulation, the motor hot spot was determined as the region of M1 cortex where the applied stimulation evoked abduction of contralateral abductor pollicis brevis using suprathreshold stimulation. Subsequently, the resting motor threshold (RMT) was defined as the least stimulation producing a motor-evoked potential (MEP) response of >50 *μ*V in at least 5 of 10 consecutive stimulations [35, 36].

If no MEP could be obtained from the affected hemisphere, the hot spot was defined as the symmetric location (mirror region) to the unaffected hemisphere [35]. Group B received HF-rTMS using the following parameters: stimulation of 20 Hz for 5 s, intertrain interval for 50 s, 20 trains, and a total of 2000 pulses at 90% RMT on the affected hemisphere [28]. Following HF-rTMS, conventional PT program, similar to group A, was administered.

Group C received CE and conventional PT treatment. For CE program, patients sat in a chair with elbows and forearms resting on a bench in front of them. The unaffected elbow was flexed to 90 degrees, and the unaffected forearm was held in mid position. For measuring maximal voluntary isometric grip strength, subjects exerted a maximal effort on grip dynamometer for five seconds. The maximal value was recorded. Three trials were performed with a two-minute rest between the trials. The mean value of three trials was recorded. During the treatment sessions, participants performed 6 sets of 5 repetitions at 60-70% maximal voluntary contraction, with a 2-minute rest between the sets (30 repetitions per session). Each repetition lasted 5 seconds followed by a 5-second recovery period. CE program and conventional PT treatment were applied in 10 sessions, 3 times a week.

In group D, HF-rTMS (similar to group B), CE (similar to group C), and conventional PT were administered.

It is worth noting that the participants performed the PT interventions with the affected side after receiving CE on the normal side.

### 2.4. Statistics

Data were analyzed using SPSS version 23 statistical package (IBM statistics, New York, USA). Baseline data of the participants were expressed as mean and standard deviation for continuous variables and median and interquartile range (IQR) for categorical variables. The Kolmogorov-Smirnov test confirmed the non-normal distribution of data. Differences between pre- and posttest values were analyzed using the Wilcoxon signed rank test. The Kruskal-Wallis test was used to analyze between-group differences. The significance level was set at *P* < 0.05.

## 3. Results

All patients completed the study protocol, and no adverse effect was reported. Demographic and baseline data of the participants are depicted in Table 1. There were no significant between-group differences in the baseline variables.

Clinical measurements before and after the interventions are presented in Table 2. Interventions significantly improved FMA-UE, BBT, grip strength, and pinch strength in all groups.

The values for FMA-UE, BBT, grip strength, and pinch strength are shown in Table 3. Statistically significant difference was observed among the groups regarding the clinical measures including FMA-UE (*H*_(2)_ = 15.348, *P* = 0.002, with a mean rank of 10.90 for group A, 17.55 for group B, 30.60 for group C, and 22.95 for group D), BBT (*H*_(2)_ = 15.348, *P* = 0.001, with a mean rank of 8.50 for group A, 21.00 for group B, 27.35 for group C, and 25.15 for group D), and grip strength (*H*_(2)_ = 15.348, *P* = 0.042, with a mean rank of 13.10 for group A, 26.20 for group B, 24.75 for group C, and 17.95 for group D); however, there was no significant difference in the pinch strength values (*H*_(2)_ = 15.348, *P* = 0.760, with a mean rank of 17.10 for group A, 22.10 for group B, 21.70 for group C, and 25.15 for group D).

Regarding pair-wise comparison between the groups, the following data were noticeable. A statistically significant difference in the FMA-UE was observed between groups A and C (*P* < 0.001), groups A and D (*P* = 0.02), and groups B and C (*P* = 0.01). A statistically significant difference was observed between groups A and B (*P* = 0.016), groups A and C (*P* = 0.0001), and groups A and D (*P* = 0.001) in term of BBT scale. Also, a statistically significant difference was seen in the grip strength between groups A and B (*P* = 0.01) and between groups A and C (*P* = 0.02).

## 4. Discussion

The FMA-UE, BBT, grip strength, and pinch strength improved significantly following 10 sessions of the interventions in all groups.

Regarding FMA-UE scores, as an indicator for upper extremity function, the groups that received CE and PT and received CE together with PT and HF-rTMS significantly improved in comparison to the control group. Also, CE+PT significantly improved FMA-UE scores in comparison to HF-rTMS+PT.

The improvement of BBT and grip strength scores was significantly greater in patients treated with HF-rTMS and PT than the control group. Moreover, patients that received CE+PT showed better improvement in the outcome measures compared to the control group except for pinch grip.

Previous studies revealed that rTMS might increase grip strength and hand dexterity in poststroke patients. Chang et al. reported that 10 daily sessions of HF-rTMS on the affected hemisphere significantly improved BBT and grip strength in patients with subacute stroke [37]. Moreover, Kim et al. reported that 10 sessions of rTMS with a frequency of 20 Hz on the ipsilateral M1 cortex significantly improved motor function of the affected upper limb in subacute phase of stroke [28]. More recent studies also are in line with our findings. Yang et al. evaluated the effects of rTMS on the affected hemisphere of patients in subacute phase of stroke. They demonstrated the short-term beneficial effects of combined rTMS and grip training protocol [38]. Luk et al. evaluated the effects of low-frequency rTMS over contralateral motor area in subacute stroke. They found that FMA-UE and BBT scores improved more in the group that received rTMs followed by 30 minutes of motor task practice than the group that received sham rTMS and the same motor task practice [16].

Noh et al. investigated the combined effects of rTMS and action observation for recovery of function in the upper extremities in subacute phase of stroke. They found that FMA score was not significantly different between the groups, while grip power significantly improved in combination group [39].

There are evidence suggesting that rTMS reduce infarct size and neural death through the upregulation of antiapoptosis of the cells located at the margin of the infarct [40–42]. HF-rTMS has been shown to induce synaptic plasticity and long-term potentiation. In addition, HF-rTMS increased corticocortical routes and improved the “rebalancing” of the cortical excitability between hemispheres via corpus callosum pathways [14]. In line with the previous studies, our findings confirmed that HF-rTMS can be used as a helpful practical adjunct to conventional PT in subacute phase of stroke. Based on a recent narrative review, rTMS should be considered as a treatment based on a functional assessment of the severity of upper extremity function like FMA-UE [43].

Our findings showed that CE+PT had led to a better functional motor recovery compared to conventional PT. The improvement of BBT, FMA-UE, and grip strength scores was significantly more pronounced in patients treated with CE than those treated with conventional PT [44, 45]. It was hypothesized that spinal circuitry is adjusted by CE. EMG studies suggested that change in the amplitude of H-reflex and V-wave following CE may be due to the changes in the intrinsic properties of Ia afferents. These studies proposed that changes in motoneuron firing rate, presynaptic inhibition, and intrinsic motoneuron properties may occur due to CE training [46, 47]. Another possibility is that supraspinal mechanisms might contribute to the contralateral strength training effect. TMS studies have demonstrated that CE can change neural drive and increase corticospinal excitability in both trained and untrained limbs. Coactivation of bilateral corticospinal pathways might incorporate bilateral central drive to the homologous muscles, since about 10% of the corticospinal fibres enter the corticospinal tract of the ipsilateral side [14]. There are callosal connections between most cortical motor areas, such as M1 cortex, supplementary motor areas, cingulate motor areas, and prefrontal areas. It has been suggested that these areas are activated during CE training. There is evidence that during CE, as well as the activity of contralateral hemisphere, the ipsilateral cortex is activated [44]. Regarding the numerous underlying mechanisms, particularly those confirming bilateral central drive following CE could lead us to understand the greater beneficial effects of CE combined with routine PT in comparison to HF-rTMS+PT on FMA-UE [48, 49]. In line with the previous studies, our findings confirmed that CE could be regarded as a supplementary therapy to conventional PT at improving motor function in patients with subacute stroke.

The results of the present study showed that the improvement of the FMA-UE and BBT scores was significantly more obvious in patients treated with rTMS+CE+conventional PT than those treated with conventional PT; however, this combination was not superior to routine PT at improving the grip strength. To the best of our knowledge, there is no study investigating the combined effects of HF-rTMS, CE, and PT on functional indices of the affected limb in patients with stroke. Du et al. evaluated the combined effects of rTMS and neuromuscular electrical stimulation (NMES) on the upper limb motor function in stroke patients with less than 3 months from the course of disease. The participants were assigned into four groups: control group, NMES group, rTMS group (frequency of 1 Hz, 20 minutes), and NMES+rTMS group. All the groups received a rehabilitation training including task-oriented occupational therapy and physiotherapy. Comparing to other groups, combining rTMS and NMES significantly improved upper extremity motor function and activities of daily life in patients with stroke. It could be surmised that combining RTMS with CE might have detrimental effects on peripheral mechanisms possibly responsible for increasing grip strength in comparison to using these methods separately.

Although all the interventions improved pinch strength more than control group, the beneficial effects were not significantly different. No study has yet evaluated the HF-rTMS on pinch strength in subacute phase of stroke. Conforto et al. showed that low-frequency rTMS improved pinch strength in a group of patients within 5–45 days poststroke with mild to severe hand paresis [50].

Also, the effects of CE on pinch grip are yet to be determined. The results of pinch might be due to basic difference of pinch and grip strength. Grip progression is most pronounced during the first 6 months poststroke and less between 6 and 12 months, while pinch force shows less progress during 6–12 months poststroke compared with hand grip force [51]. Since our participants were in the subacute phase of stroke, no significant difference was observed among the interventions regarding pinch strength.

## 5. Limitations of Study

Some limitation of the present study should be noted. First, all patients in this study were in subacute phase of stroke (within 6 months of onset). The efficacy of the interventions should also be evaluated in the chronic phase of stroke, particularly on the outcomes requiring more time for progression such as pinch strength. Second, the participants had a score of 22-44 points on the FMA-UE. It is necessary to examine these protocols on patients with more severe hand deficit.

Moreover, we did not use a neuroimaging tool, such as fMRI or positron emission tomography to confirm our clinical findings. Therefore, we suggest using these assessments to provide more complementary information on the underlying mechanisms of the protocols. It is worth noting that as we were to evaluate the effects of the intervention in the subacute phase of stroke, no follow-up period could be considered for the study. Also, the number of the participants in each group seems to be low. Future studies with large number of patients are warranted to verify our findings.

## 6. Conclusion

The present study proposed that clinicians should select the therapeutic methods more specifically in accordance with their goal. For instance, when the goal is to improve upper extremity function, CE+PT was significantly more effective than HF-rTMS+PT. Also, CE+PT and HF-rTMS+PT were more effective than CE+RTMS+PT at improving the grip strength. Therefore, combining several methods would not always provide better results.

## Figures and Tables

**Table 1:** Demographic and baseline data of patients in the four groups (*n* = 40).

Parameters	Group A (*n* = 10)	Group B (*n* = 10)	Group C (*n* = 10)	Group D (*n* = 10)	*P* value
Age (years)	53.90 ± 13.06	50.50 ± 9.47	59.70 ± 5.65	50.00 ± 11.10	0.14
Sex, male/female, *n* (%)	5 (50)/5 (50)	6 (60)/4 (40)	7 (70)/3 (30)	5 (50)/5 (50)	0.77
Onset time (months)	3.20 ± 1.68	3.00 ± 1.41	2.30 ± 1.41	2.80 ± 0.91	0.52
Dominant hand, right/left, *n* (%)	10 (100)/0 (0)	8 (80)/2 (20)	10 (100)/0 (0)	10 (100)/0 (0)	0.09
Affected hand, right/left, *n* (%)	5 (50)/5 (50)	4 (40)/6 (60)	4 (40)/6 (60)	4 (40)/6 (60)	0.95
Treatment duration (sessions)	28.00 ± 27.40	38.00 ± 16.19	24.50 ± 20.06	27.90 ± 11.33	0.46
FMA-UE score	36.7 ± 7.71	30.5 ± 6.78	33.00 ± 6.99	33.8 ± 6.40	0.28
BBT (unit)	23.2 ± 16.47	13.5 ± 10.9	14.7 ± 9.12	17.10 ± 11.51	0.31
Grip strength (kg)	4.67 ± 3.89	5.27 ± 2.39	5.25 ± 5.63	7.39 ± 6.56	0.62
Pinch strength (kg)	5.44 ± 2.97	2.55 ± 1.33	2.40 ± 1.45	2.75 ± 1.52	0.82

Abbreviations. FMA-UE: Fugl-Meyer assessment for upper extremities; BBT: box and block test. Values demonstrate mean (standard deviation) or frequency. Study groups were as follows: (A) physiotherapy, (B) HF-rTMS+physiotherapy, (C) cross education+physiotherapy, and (D) HF-rTMS+cross education+physiotherapy.

**Table 2:** Clinical measurements of the affected upper limb at pre- and postintervention in the four groups.

	Pre (median)	Post (median)	Change rate (median)	*Z*-score	*P* value
FMA-UE (score)					
Group A	40.00	46.00	5.00	-2.807	0.005
Group B	32.00	38.00	9.00	-2.812	0.005
Group C	32.00	52.00	21.00	-2.805	0.005
Group D	33.00	47.5	14.50	-2.673	0.008
BBT					
Group A	23.50	25.00	2.00	-2.687	0.007
Group B	8.00	15.50	7.00	-2.810	0.005
Group C	12.00	19.50	9.00	-2.821	0.005
Group D	15.00	29.00	8.50	-2.668	0.008
Grip strength (kg)					
Group A	2.72	5.21	1.20	-2.226	0.026
Group B	4.54	7.39	2.86	-2.814	0.005
Group C	2.65	7.40	3.56	-2.8.3	0.005
Group D	4.31	5.67	1.58	-2.807	0.005
Pinch strength (kg)					
Group A	2.72	3.55	0.91	-2.371	0.018
Group B	1.81	2.93	1.01	-2.807	0.005
Group C	2.57	3.55	1.13	-2.668	0.008
Group D	2.27	3.25	0.98	-2.829	0.005

FMA-UE: Fugl-Meyer assessment for upper extremities; BBT: box and block test. Study groups were as follows: (A) physiotherapy, (B) HF-rTMS+physiotherapy, (C) cross education+physiotherapy, and (D) HF-rTMS+cross education+physiotherapy.

**Table 3:** Comparison of clinical measurements among the four different groups using the Kruskal-Wallis test.

	Mean rank	Chi-square	*P* value
FMA-UE (score)		15.348	0.002
Group A	10.90		
Group B	17.55		
Group C	30.60		
Group D	22.95		
BBT		15.761	0.001
Group A	8.50		
Group B	21.00		
Group C	27.35		
Group D	25.15		
Grip strength (kg)		8.213	0.042
Group A	13.10		
Group B	26.20		
Group C	24.75		
Group D	17.95		
Pinch strength (kg)		1.172	0.760
Group A	17.10		
Group B	22.10		
Group C	21.10		
Group D	21.70		

Abbreviations: FMA-UE: Fugl-Meyer assessment for upper extremities; BBT: box and block test. Values demonstrate difference values of mean rank. Study groups were as follows: (A) physiotherapy, (B) HF-rTMS+physiotherapy, (C) cross education+physiotherapy, and (D) HF-rTMS+cross education+physiotherapy.

## Data Availability

The data that support the findings of this study are available on request from the corresponding author.
